# Research Progress on Construction Technology of 3D Human Skin Models and Its Application Prospects in Dermatology

**DOI:** 10.3390/ijms27041808

**Published:** 2026-02-13

**Authors:** Liping Li, Wenqi Si, Zhehu Jin, Wenyu Jin

**Affiliations:** Department of Dermatovenereology, Affiliated Hospital of Yanbian University, Yanji 133000, China; leelyupyung@163.com (L.L.); 19941063569@163.com (W.S.)

**Keywords:** 3D human skin models, tissue engineering, bioprinting, dermatology, disease modeling, precision medicine, multi-omics

## Abstract

Traditional two-dimensional (2D) cell cultures and animal models often fail to replicate the complex layered structure and pathological microenvironment of human skin. This paper systematically reviews the latest advancements in three-dimensional (3D) human skin model construction, specifically focusing on bioprinting, organoid, and organ-on-a-chip technologies. Our analysis highlights that the integration of vascularization, skin appendages, and multi-omics data represents the core advancements in enhancing physiological realism for simulating. Despite these strides, challenges such as a lack of standardization and high medical costs remain as barriers to clinical translation. We conclude that future directions must leverage interdisciplinary synergy—integrating artificial intelligence and personalized multi-omics—will be essential to transition 3D skin models from laboratory tools to precise clinical applications.

## 1. Introduction

Skin diseases represent a staggering global health burden, affecting nearly one-third of the world’s population and ranking as the fourth leading cause of non-fatal disease burden worldwide [1]. Beyond physical discomfort, conditions such as chronic wounds, melanoma, and inflammatory dermatoses impose a severe psychological and economic toll on society. Despite their prevalence, human skin acts as a critical homeostatic barrier. Its intricate structure makes dermatological research uniquely challenging. Traditional research often relies on 2D cell cultures or animal models. However, 2D models lack realistic barrier properties, while animal studies involve high costs and ethical controversies [2]. These limitations are particularly evident when simulating complex pathologies like wound healing or skin tumorigenesis (Table 1). The irritation and functional impairment caused by skin diseases underscore the urgent need to develop more advanced research models [3,4]. As understanding of skin physiology and pathological mechanisms deepens, researchers are calling for novel model systems that accurately recapitulate the 3D structure and heterogeneity of human skin.

The rapid development of computational technologies and bioengineering is driving a paradigm shift in dermatological research. Advanced 3D skin models utilize bionic construction techniques to replicate the layered structure and cellular heterogeneity of native skin [5]. By integrating complex cell–cell and cell–matrix interactions, these models more accurately mimic human biological responses. Consequently, they provide a platform for generating clinically relevant data. These advancements offer new pathways for precision medicine and the development of optimized therapeutic strategies [6].

This review comprehensively examines research advances in core construction technologies of 3D human skin models for dermatological applications, disease-specific modeling in dermatopathology, and key challenges in clinical translation, while outlining future directions for advancing precision dermatology. Firstly, it systematically outlines key developments in three core construction technologies: bioprinting technology, organoid technology, and organ-on-a-chip technology. Secondly, it elaborates on disease-specific modeling achievements for various dermatological conditions, including pathological simulations of wound healing models, skin cancer models, inflammatory skin disease models, aging skin models, and skin infection models. Finally, it deeply analyzes challenges hindering clinical translation—lack of standardized protocols, bottlenecks in simulating pathological complexity, regulatory/ethical gaps, and cost–effectiveness imbalances. Integrating interdisciplinary collaboration trends, it proposes leveraging multi-omics data and artificial intelligence to drive 3D skin models toward personalized medicine and closed-loop therapeutic regimens. This framework aims to provide references for transforming dermatological research paradigms and implementing precision medicine.

## 2. Advances in 3D Skin Model Construction Technologies

### 2.1. Bioprinting Technology

3D bioprinting technology, an advanced computer-controlled manufacturing technique, enables the fabrication of precise 3D structures layer-by-layer deposition of biological factors and bioinks, opening up new avenues for skin tissue engineering and regenerative medicine [7]. This technology exhibits unique advantages in the field of skin tissue engineering, enabling precise control over the spatial distribution of multiple components and structural complexity [4]. Current research focuses include improvement of bioink formulations, full-thickness skin printing techniques, vascularization strategies, and printing of skin appendages [8]. In particular, composite scaffold materials based on sodium alginate (SA) and gelatin (Gel) have been successfully applied in the manufacturing of artificial skin [9]. Bioprinting technology not only rapidly produces models for high-throughput anticancer drug testing but also customizes skin substitutes with specific shapes to meet clinical and industrial requirements [10,11].

Extrusion-based, droplet-based, laser-based, and light-based bioprinting methods are utilized for precise deposition of cells and biomaterials to mimic the complex structure of skin. While various bioprinting strategies exist, they differ fundamentally in their dispensing mechanisms. Extrusion-based bioprinting dispenses continuous filaments of high-viscosity bioinks using pneumatic or piston-driven forces. It is the most preferred method for fabricating clinically relevant sized skin substitutes due to its scalability, although the shear stress at the nozzle tip poses a risk to cell viability. In contrast, droplet-based bioprinting generates discrete droplets via thermal or piezoelectric actuators. It offers superior resolution for precise keratinocyte patterning but is limited to low-viscosity bioinks, which often lack structural fidelity for full-thickness models. Laser-assisted bioprinting utilizes laser pulses to propel cell-laden droplets onto a substrate. LAB achieves the highest cell viability by avoiding nozzle-induced shear stress, making it ideal for arranging sensitive melanocytes or stem cells, though its high cost and slow fabrication speed limit its use in high-throughput production [8,12]. These methods permit precise spatial arrangement of programmed cells, facilitating the printing of skin equivalents either within bioprinters or directly at wound sites. Additionally, 3D bioprinting enables the construction of full-thickness skin models, such as those achieved through collagen hydrogel-based structures that recapitulate the skin’s cellular microenvironment to create composite models incorporating the epidermis, dermis, and hypodermis. This capability bridges the structural and functional gap between natural and artificial skin [4,8,13,14].

The optimization of bioinks represents a core advancement in the construction of functional skin models, primarily involving the formulation and application of novel materials. Hydrogel-based bioinks, such as gelatin methacrylamide (GelMA), sodium alginate, and gelatin, are widely used because they can mimic the mechanical properties of skin and support cellular viability [15]. For example, ionic conductive bioinks have been developed to fabricate bilayer ionic conductive skin scaffolds, enhancing electrical signal conduction capabilities. Researchers are actively developing composite bioinks, such as scaffolds combining sodium alginate and gelatin, to improve pore structure and physicochemical properties, thereby promoting angiogenesis. These bioinks enhance the flexibility and biocompatibility of printed constructs, addressing printability issues associated with traditional materials like conductive hydrogels [9,16].

Significant advancements have been achieved in model construction with bioprinting technology to realize skin multifunctionality and fidelity. 3D bioprinting enables the construction of full-thickness skin equivalents, encompassing the epidermis, dermis, and hypodermis, with a particular focus on addressing vascularization [14,17]. By creating perfusable vascular networks that recapitulate the skin’s superficial vascular plexus, this supports immune cell circulation and the wound healing process. This contributes to bridging the gap in vascularization strategies, offering physiologically relevant models for wound repair [18,19].

Bioprinting technology demonstrates unique advantages in skin engineering. Its high resolution, customizability, and reproducibility allow for the precise replication of skin shape and structure, enabling rapid and reliable bionic skin production [11]. Concurrently, this technology offers flexibility, facilitating the direct printing of skin at the wound site, thereby reducing adverse reactions and complications associated with traditional surgical methods [20]. Overall, it propels advancements in tissue engineering, providing a novel foundation for skin regeneration, drug development, and translational applications. However, bioprinted skin models still face challenges. The main limitations include the inadequacy of vascularization strategies, the functional integrity of skin appendages, and the matching of mechanical properties to those of native skin. For instance, existing technologies fail to fully replicate the sensory, regenerative, and immune functions of the skin [21]. Future research needs to focus on optimizing bioinks, dynamic bioprinting (such as 4D printing), and integrating artificial intelligence to enhance the physiological authenticity of the models [20,22].

In summary, 3D bioprinting offers unparalleled precision in the spatial arrangement of cells and matrix components, enabling the reproducible fabrication of patient-specific, full-thickness skin constructs with customized architectures. This technology is particularly advantageous for creating geometrically complex grafts and facilitating in situ repair. However, significant challenges persist, particularly regarding the successful integration of functional vascular networks and nerves, as well as the need to optimize bioink formulations to better match the mechanical integrity and long-term stability of native human skin.

### 2.2. Organoid Technology

Organoid technology represents a breakthrough innovation in the life sciences, providing a revolutionary platform for dermatological research. The construction of organoid technology in skin models relies on multidisciplinary engineering strategies, enabling the evolution of skin models from simple multilayered structures to highly complex organoids that more closely resemble native skin. Early skin models were primarily layered epidermis or dermis, whereas recent advances support the generation of appendage-bearing, cyst-like skin organoids [23]. Furthermore, some models have successfully replicated the epidermis, dermis, and appendage structures such as sweat glands, enhancing their precision in physiological simulation [24].

Organoid models achieve more comprehensive functional simulation by integrating key components such as immune cells and vasculature. Specifically, 3D skin organoids support skin regeneration processes, including epithelialization, vascularization, and suppression of excessive inflammation, as validated in wound healing experiments [14,25,26]. Skin organoids can model disease heterogeneity and facilitate research on cancer invasion dynamics and drug responses, enabling visualization of disease progression through high-resolution imaging and analysis. They are widely used to study skin development mechanisms and diseases [27]. Additionally, they provide personalized models tailored to specific patient genetics or disease profiles, which proves particularly valuable for exploring human-specific disease mechanisms [23,28,29]. Furthermore, organoid models play a pivotal role in drug development, enabling toxicity testing and pharmacodynamic evaluation while circumventing ethical concerns associated with animal experimentation [30].

Despite significant advancements in organoid technology, current models still face challenges in complexity, maturation, and microenvironment control (Table 2). For instance, the differentiation process of skin organoids may result in heterogeneous cell populations, compromising model reliability. Additionally, the physiological relevance of these models has not yet fully achieved native skin levels, such as lacking complete neural or sensory functions. Future research will focus on precise microenvironment control, while integrating organ-on-a-chip technology and single-cell analysis is expected to overcome existing limitations and drive the application of organoids in skin research and therapeutics [24,27,31].

The generation of skin organoids is fundamentally driven by the intrinsic self-organization capabilities of human induced pluripotent stem cells (hiPSCs). Unlike scaffold-based models, organoid formation relies on the precise temporal modulation of developmental signaling pathways to mimic embryonic skin development steps. Under specific stepwise chemical induction within a 3D matrix, these cells undergo symmetry breaking and self-assemble into cyst-like structures. Crucially, a ‘dual-SMAD’ inhibition strategy is often employed to suppress neural lineage differentiation and promote epidermal specification. Advanced protocols further involve an air–liquid interface (ALI) culture phase after the initial cyst formation, which is essential for driving the stratification of the epithelium and the maturation of cornified envelopes, closely mirroring the in vivo epidermal barrier formation [23,31].

Collectively, skin organoids excel in recapitulating complex developmental processes and preserving donor-specific genetic heterogeneity, making them ideal platforms for modeling congenital disorders and personalized drug responses. They uniquely capture the self-organization capabilities of skin tissues. Nevertheless, their broader application is currently constrained by stochastic variability in organoid formation, the presence of heterogeneous cell populations, which complicates standardization, and the lack of defined systemic interactions, such as immune circulation and neural innervation, required for complete physiological mimicry.

### 2.3. Organ-on-a-Chip

Organ-on-a-Chip (OOC) technology achieves precise simulation of the skin microenvironment by employing microfluidic systems and 3D cell culture methods, resulting in significant advancements in constructing 3D human skin models. Compared to traditional 2D or static 3D models, this technology more accurately replicates the physiological structure, functions, and dynamic processes of human skin—including vascularization, signaling pathways, and drug responses—thereby providing an efficient platform for drug screening, disease modeling, and toxicity testing. A recent study developed a full-thickness perfused skin-on-a-chip and demonstrated that TNF-α-induced inflammation and dexamethasone treatment yielded cytokine response profiles consistent with human skin biopsies, highlighting the model’s ability to mimic in vivo drug responses [32].

Vascularization is one of the core advances in organ-on-a-chip technology, enhancing the physiological relevance of skin models. Vascularized 3D skin models achieve dynamic perfusion through microfluidic devices, enabling precise control over blood flow, oxygen concentration, and nutrient distribution. This replicates key physiological processes of human skin, such as multicellular signaling pathways and endothelial barrier functions, thereby more realistically recapitulating the skin microenvironment. The vascular network in skin-on-a-chip models supports the circulation of immune cells, including T-cell migration, providing a tool for studying skin immunology, skin pathology, skin physiology, and inflammatory responses [18,24,33,34]. Organ-on-a-chip platforms address the limitations of traditional static skin models in nutrient transport and waste clearance by introducing microfluidic channels to achieve dynamic culture conditions, offering a framework for organ-on-a-chip models of multiple tissues [35,36,37].

Physically, skin-on-a-chip devices typically utilize polydimethylsiloxane (PDMS) due to its high oxygen permeability and optical transparency, which allows for real-time microscopic imaging. The architecture generally follows a ‘sandwich’ design: an upper chamber maintaining the epidermis at the air–liquid interface, separated by a semi-permeable porous membrane from a lower microfluidic channel. This lower channel is continuously perfused with culture medium via syringe pumps or peristaltic pumps to mimic blood flow. A critical technical advantage of this setup is the generation of controlled fluid shear stress on the endothelial cells lining the lower channel. This mechanical stimulus is physiologically vital for maintaining endothelial barrier integrity and preventing vascular leakage, a feature entirely absent in static Transwell inserts [33,35].

Organ-on-a-chip technology integrates advanced manufacturing methods, namely 3D bioprinting, to create skin models with high precision and complex structures. The model contains a perfusable vascular network resembling the superficial vascular plexus of the skin and closely surrounding bioengineered hair follicles, enabling dynamic simulation of immune cell circulation, which significantly enhances the functional maturity of skin tissue [18,38]. 3D printing not only simplifies the fabrication of microfluidic chips but also improves the spatial organization of skin models through a layering and stacking process, making the models more closely approximate the anatomical structure of real skin [39].

Organ-on-a-chip skin models have become a key platform for drug screening, playing a vital role in reducing costs and minimizing animal experiments. Organ-on-a-chip technology enhances the function of traditional static skin models through dynamic conditions, enabling efficient evaluation of the toxicity, efficacy, and delivery efficiency of drug candidates in the pharmaceutical industry [35,40]. Additionally, the skin-on-a-chip platform provides a non-invasive approach for testing drugs and modeling skin diseases, facilitating the development of diagnostic or therapeutic strategies, particularly well-suited for high-throughput screening scenarios. In drug evaluation, these models allow for more accurate monitoring of inflammatory mediators and pharmacodynamic responses, enabling the analysis of tumor microenvironments and the development of targeted therapies [3,38]. However, organ-on-a-chip skin models exhibit limitations in complexity and maturity. Current models struggle to fully replicate physiological dynamics such as sensory functions and regenerative capacities of the skin, while facing challenges related to the reliability of microfluidic devices [24]. Furthermore, addressing obstacles in miniaturization and automation is essential to achieve scalable application [41,42]. In summary, organ-on-a-chip technology has achieved critical breakthroughs in constructing 3D human skin models, significantly improving the physiological relevance and utility of skin models, and continued efforts to overcome challenges in standardization and environmental control will drive this technology to become a core tool for drug testing and disease research.

Overall, organ-on-a-chip platforms significantly advance the physiological relevance of in vitro models by introducing dynamic fluid flow and mechanical cues that closely mimic the systemic microenvironment and vascular perfusion. This capability allows for precise control over nutrient delivery and waste removal, which is critical for drug toxicity testing. Despite these benefits, the widespread adoption of these systems is hindered by the technical complexity of microfluidic fabrication, issues with device reliability and leakage, and the challenges associated with scaling up these intricate systems for high-throughput industrial applications.

## 3. Advances in Disease-Specific Modeling Research

This section elaborates on the progress of 3D skin models in simulating specific dermatological conditions. To provide a concise overview, Table 3 summarizes the representative models, construction strategies, and key innovations across various disease categories discussed in the following subsections.

### 3.1. Construction of Wound Healing Models

Wound repair represents a critically urgent area for clinical breakthroughs. Wound healing models constitute a vital direction within 3D skin model research. Conventional models, however, remain insufficient: 2D cell cultures fail to recapitulate the 3D physiological microenvironment of the skin, rendering them incapable of simulating synergistic cellular interactions [2,36,63,64,65]; animal models exhibit significant translational limitations due to interspecies disparities in healing mechanisms, hindering the efficiency of translating basic research into clinical applications [2,66]. Constructing full-thickness skin wound models via 3D bioprinting technology enables precise matching of wound size and shape, thereby providing a standardized platform for evaluating repair efficacy.

**Figure 1:**
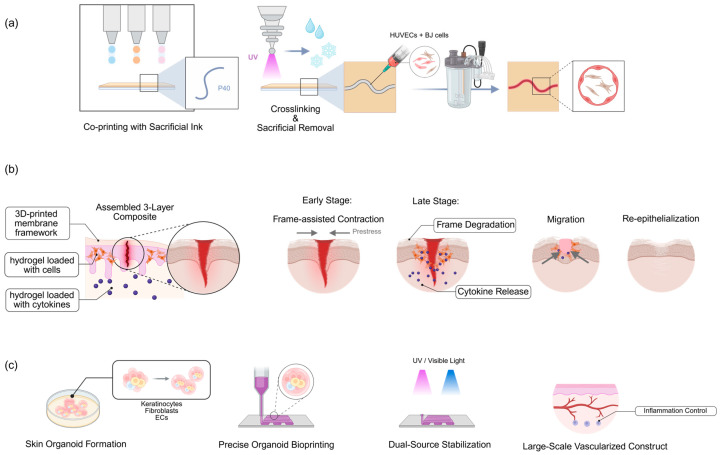
Advanced 3D bioprinting strategies for wound healing and skin regeneration. (**a**) Construction of a perfusable vascularized skin model (based on Maggiotto et al. [43]): utilizing multi-material bioprinting with sacrificial P40 ink to create hollow vascular lumens for HUVEC seeding and perfusion. (**b**) Spatiotemporally controllable 3-layer biomimetic model (based on Chen et al. [44]): featuring a top 3D-printed membrane for contraction, a middle cell-loaded hydrogel for filling, and a bottom cytokine-loaded base for sustained signaling. (**c**) Integration of skin organoids with bioprinting (based on Zhang et al. [25]): precise arrangement of organoid spheres using dual-source stabilization to promote accelerated re-epithelialization and vascularization.

The effective integration of vascular systems remains a critical challenge limiting the physiological relevance and clinical utility of 3D bioprinted skin models throughout their development. This requirement stems from the need to ensure adequate nutrient and oxygen distribution within skin models to mimic the physiological environment of native skin, thereby preventing tissue necrosis and supporting cellular viability. To address this challenge while addressing the therapeutic needs of severe skin injuries, Maggiotto et al. utilized multicellular synergy and multi-material bioprinting to construct a perfusable 3D vascularized skin model (Figure 1a) [43]. Neonatal foreskin-derived fibroblasts (BJ cells) and human epidermal keratinocytes (HEK cells) were selected to form the dermal and epidermal layers, respectively, while human umbilical vein endothelial cells (HUVECs) were incorporated to enable vasculogenesis. For bioink design, gelatin methacryloyl (GelMA) served as the structural matrix for both dermal and epidermal layers, with Pluronic F127 (P40) employed as a sacrificial material to print temporary vascular channels (Figure 1a). During the printing process, a multi-material extrusion-based bioprinter was used to sequentially fabricate vascular channels in a serpentine configuration, followed by the dermis and epidermis. Following UV-induced crosslinking and solidification of GelMA, low-temperature treatment was applied to remove P40, resulting in hollow vascular lumens. Subsequently, HUVECs and BJ cells (70:30 ratio) were seeded into the channels and cultured under continuous perfusion using a customized bioreactor. Results demonstrated that endothelial cells successfully lined the luminal surfaces, forming barrier-functional vascular structures. The model achieved spontaneous wound closure over a 14-day culture period, confirming its vascular perfusion capacity and regenerative potential. This study not only surmounts the technical bottleneck of vascular integration but also enhances clinical compatibility through its “perfusable” design, establishing a foundation for future wound-healing applications.

Building on vascularized models, Chen et al. have further focused on dynamically simulating the wound healing process. They developed a spatiotemporally controllable biomimetic skin model tailored to the requirements of different healing stages, as illustrated in Figure 1b [44]. This model comprises three layers: the top layer is a 3D-printed membrane framework, the middle layer is a hydrogel loaded with cells, and the bottom layer is a hydrogel loaded with cytokines. During the initial phase of wound repair, the membrane framework in the top layer assists directly in wound edge contraction through prestress, thereby accelerating closure. As the repair progresses, the membrane framework gradually degrades, enabling cells from the middle-layer hydrogel to migrate along the residual membrane structure to the wound site for tissue filling. Concurrently, the bottom-layer hydrogel slowly releases cytokines to provide sustained nutritional support and signaling regulation throughout the entire repair process. In validation using rat full-thickness skin injury models, this biomimetic skin not only significantly shortened wound closure time but also promoted granulation tissue formation and epithelial regeneration, demonstrating its suitability and value for dynamic repair processes (Figure 1b).

In addition to innovative structural design, material modification and printing technology optimization have emerged as crucial directions for improving the performance of 3D skin models. Choi et al. utilized digital light processing (DLP) 3D printing technology with a composite bioink composed of methacrylated silk fibroin (Silk-GMA) and Gel-GMA to construct a full-thickness skin model exhibiting both mechanical stability and biocompatibility [45]. In their study, the team seeded keratinocytes, fibroblasts, and vascular endothelial cells onto the corresponding structures to recapitulate the synergistic interactions among the epidermis, dermis, and vasculature. Long-term culture results demonstrated that the model maintained structural integrity without significant degradation over a period exceeding four weeks, while cellular viability remained at high levels. Further experiments confirmed that the introduction of epidermal growth factor (EGF) significantly enhanced the epidermal regeneration rate and dermal repair quality. This indicated the model’s adaptability in modulating growth factor signaling to meet the repair requirements of diverse wounds, thereby expanding the application scope of artificial skin models in wound mechanism research and drug screening. Similarly, Xu et al. developed a 3D skin model with anti-inflammatory functionality by incorporating halloysite nanotubes (HNT) into a collagen–alginate–hyaluronic acid composite hydrogel [46]. The addition of HNT not only reinforced the mechanical strength of the hydrogel but also facilitated keratinocyte differentiation and fibroblast adhesion through its unique nanostructure. Concurrently, it regulated the release of inflammatory factors, promoting wound healing while minimizing scar formation. This approach provides a novel experimental tool for investigating skin inflammation mechanisms and advancing trauma repair strategies.

With the integrated development of technology, the combination of skin organoids with 3D bioprinting offers a more groundbreaking strategy for treating large-area skin defects. Zhang et al. utilized human keratinocytes, fibroblasts, and endothelial cells to induce the formation of skin organoid spheres, which were then precisely arranged using extrusion-based bioprinting technology (Figure 1c) [25]. These spheres were solidified using dual-photo source cross-linking technology to ensure structural stability. As a result, this organoid-integrated model has been shown to significantly accelerate the healing process of extensive wounds through multiple mechanisms, including in situ regeneration, accelerated epithelialization, spontaneous formation of microvascular networks, and suppression of excessive inflammation (Figure 1c). These capabilities effectively overcome the limitations of “low repair efficiency and inadequate inflammatory control” associated with conventional models, offering novel approaches for the clinical management of extensive burns and traumatic wounds.

In summary, defining a high-fidelity 3D wound healing model requires satisfying specific physiological criteria beyond simple structural mimicry. Based on the advancements reviewed above, the minimum requirements for a robust model include: (1) a functional vascular network to support long-term tissue survival and facilitate immune cell infiltration [43]; (2) spatiotemporal dynamic controllability to accurately recapitulate the sequential phases of hemostasis, inflammation, proliferation, and remodeling [44]; (3) mechanical stability matching native tissue elasticity to withstand wound contraction forces without premature degradation [45]; and (4) multicellular complexity, incorporating skin appendages and immune regulation to distinguish between true regenerative healing and fibrotic scar formation [25,46]. Fulfilling these criteria is essential for transitioning these models from descriptive morphological tools to predictive preclinical platforms for therapeutic evaluation.

### 3.2. Simulation of Skin Tumor Microenvironment

#### 3.2.1. Melanoma

In the field of skin tumor research, 3D skin models have emerged as a core tool for dissecting the pathological mechanisms of malignant skin diseases such as melanoma, due to their high fidelity in replicating the in vivo microenvironment. Compared to traditional 2D culture systems or simple tumor spheroid models, 3D skin models integrating the tumor microenvironment not only more accurately recapitulate the invasive phenotypes of melanoma but also precisely capture its drug resistance characteristics. This provides an indispensable research platform for uncovering key mechanisms of tumor progression and identifying potential therapeutic targets [67,68].

**Figure 2:**
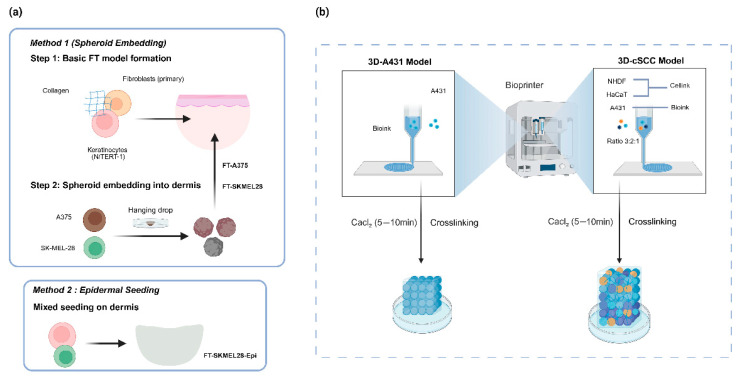
3D models for studying the skin tumor microenvironment. (**a**) Construction strategies for melanoma models based on Daugaard et al. [47]: Method 1 (Spheroid Embedding) involves incorporating tumor spheroids into a dermal matrix, while Method 2 (Epidermal Seeding) mimics tumor progression via mixed cellular seeding on the dermis. (**b**) 3D bioprinting workflow for cutaneous squamous cell carcinoma (cSCC) according to Kurzyk et al. [50], comparing the multicellular 3D-cSCC model with the single-cell 3D-A431 model for drug efficacy and interaction analysis.

Daugaard et al. addressed critical needs in melanoma microenvironment simulation by developing a full-thickness (FT) 3D co-culture model incorporating multiple cell types [47]. This system incorporates hTERT-immortalized N/TERT-1 keratinocytes as the primary epidermal layer cells and primary human dermal fibroblasts as the dermal matrix support cells, while introducing two melanoma cell lines—A375 and SK-MEL-28—to recapitulate the characteristics of different tumor subtypes. As illustrated in Figure 2a, two distinct construction strategies were developed to simulate various tumor microenvironments. The first construction protocol (Method 1: Spheroid Embedding) involves a two-step process: fibroblasts are initially mixed with a collagen matrix, and keratinocytes are seeded to form a basic full-thickness skin model. Subsequently, melanoma cells are induced to self-aggregate into tumor spheroids via the hanging drop method, which are then embedded into the dermal layer of the model. This approach yields two specific melanoma models: FT-A375 and FT-SKMEL28 (Figure 2a). The second construction method (Method 2: Epidermal Seeding) entails mixing A375 or SK-MEL-28 cells with keratinocytes and directly seeding them onto the surface of the dermal layer, resulting in the FT-SKMEL28-Epi model. Research data demonstrate that melanoma cells in this system stably retain their inherent biological properties, with significant upregulation of invasion-related gene expression levels. In drug testing, the BRAF inhibitor vemurafenib effectively reduces melanoma spheroids within the model, confirming its potential for anticancer drug screening. However, this model has certain limitations: in the FT-SKMEL28-Epi model, melanoma cells disrupt epidermal integrity, limiting its use for studying epidermal invasion processes; additionally, A375 cells frequently induce model contraction, leading to abnormal tissue architecture, thus rendering them unsuitable for epidermal co-culture scenarios.

Sandri et al. developed two complementary 3D models for melanoma drug resistance research, respectively focusing on “simple invasion behavior assessment” and “complex tissue microenvironment simulation” [48]. The first model constructs melanoma spheroids within a collagen matrix using the liquid overlay technique. Quantitative analysis revealed that drug-resistant melanoma cells exhibited a significantly higher invasion index compared to non-resistant cells. While this model enables rapid assessment of cellular invasive ability and recapitulates the impact of the 3D microenvironment on cell behavior, it has limitations including insufficient simulation of the dermal microenvironment by the collagen matrix and subjective dependence in invasion index quantification. The second model optimized tissue structure through multicellular co-culture: melanoma cells were mixed with keratinocytes while incorporating melanocytes to restore the physiological composition of skin. Using an air–liquid interface culture method, multilayered skin tissue was formed. Results demonstrated that non-resistant melanoma cells formed invasion foci with clear boundaries, whereas resistant cells could break through the epidermal layer into the dermal region. Further molecular mechanism analysis identified a significant increase in matrix metalloproteinase-2 (MMP-2) expression levels within the drug-resistant model. The strength of this model lies in its coverage of multi-scale research needs—from single-cell invasion to tissue-level interactions—integrating molecular detection, cellular behavior observation, and extracellular matrix analysis. This comprehensive approach elucidates the invasive mechanisms of drug-resistant cells, not only providing a multidimensional platform for BRAF inhibitor resistance research but also furnishing experimental evidence for developing MMP-2-targeted combination therapies.

Massaro et al. also adopted a dual-model strategy to investigate the regulation of melanoma invasion capabilities and evaluate drug responses [49]. The first model utilized agar-coated culture plates to induce melanoma cells to form tumor spheroids. These spheroids were subsequently embedded in bovine collagen type I gel and treated with 2-Methoxyestradiol (2-ME). The results demonstrated that 2-ME significantly inhibited the invasion capability of melanoma cells, validating the applicability of this model for screening the anti-invasive activity of small-molecule compounds. The second model further optimized microenvironmental fidelity: SK-Mel-28 melanoma cells were co-cultured with keratinocytes and normal melanocytes on a collagen–fibroblast composite matrix. This co-culture first established a complete epidermal layer, which was then transferred to an air–liquid interface for cultivation, ultimately yielding a 3D skin model more closely resembling the physiological state. The core advantages of this model are that on one hand, it allows precise assessment of the invasion pathways, migratory capabilities, and interactions with stromal cells. On the other hand, it can simulate the drug penetration through skin tissue and the differences in drug resistance of tumor cells. This system provides an experimental platform better aligned with clinical scenarios for subsequent research on optimizing drug delivery efficiency and investigating resistance mechanisms.

#### 3.2.2. Squamous Cell Carcinoma (cSCC)

In the research field of cutaneous squamous cell carcinoma (cSCC), the 3D human skin model has achieved significant progress due to its advantage in more realistically simulating the cSCC microenvironment and drug responses. This model provides a powerful and reliable tool for mechanistic studies, drug screening, and personalized therapy of cSCC. Among these, Kurzyk et al. successfully constructed a cSCC-specific model using 3D bioprinting technology (Figure 2b) [56]. This model not only encompasses the 3D structure of normal skin but also incorporates a multicellular system for cSCC, accurately replicating the key characteristics of cSCC. To evaluate the performance of this model comprehensively, the research team conducted multi-dimensional comparative analysis. They compared the bioprinted 3D-cSCC model—composed of HaCaT keratinocytes, primary normal human dermal fibroblasts, and A431 cancer cells (three-cell component)—with three other models: the bioprinted 3D-A431 model (consisting solely of A431 cancer cells, single-cell component), A431 cancer cell spheroids, and the conventional 2D model (Figure 2b). Simultaneously, functional assessment was performed using cetuximab (CTX), combined with the MTS assay to validate model reliability. This study elucidated the potential of this 3D model in better understanding the complex interactions of cellular components within the tumor microenvironment, thereby establishing a critical platform for drug screening and personalized therapy of cSCC.

In addition to the aforementioned cSCC-specific models, researchers have developed full-thickness models (FTMs), which integrate multiple cell types to directly mimic the tumor microenvironment of cSCC. These models facilitate the elucidation of behavioral patterns of relevant cells in a 3D context and enable an in-depth investigation into the anti-tumor mechanisms of therapeutic agents, thereby providing robust experimental evidence for clinical research. Notably, the contributions of He et al. in this area are particularly significant, as they established a 3D model that effectively simulates the role of cancer-associated fibroblasts (CAFs) in cSCC [51]. Specifically, the research team utilized the relevant cell types to develop a 3D full-thickness model of SCC on a dermal matrix containing cancer-associated fibroblasts (CAFs). On this model, they evaluated the anti-tumor effects of drug-loaded nanoparticles via two routes: topical administration and intradermal injection. Experimental outcomes demonstrated that the drug-loaded nanoparticles exhibited potent anti-tumor activity against cSCC cells within the 3D model. They could further enhance therapeutic efficacy by modulating the function of cancer-associated fibroblasts (CAFs), and this finding uncovers the broad application potential of this 3D model in future studies on cSCC pathogenesis.

### 3.3. Inflammatory Skin Disease Models

#### 3.3.1. Psoriasis

In psoriasis pathology and drug development, 3D skin equivalents (HSEs) have become a critical tool bridging basic research and clinical applications due to their ability to recapitulate the in vivo microenvironment. Scheurer et al. achieved a breakthrough in psoriatic pathological modeling—from immune-free to immune-competent—through the design of two core human skin equivalents (HSEs), laying a fundamental framework for subsequent model optimization [52]. The team initially developed two types of basic immune-free human skin equivalents (HSEs), and adjusted matrix components and construction protocols to meet the needs of different research cycles. The first type is fibroblast-derived matrix HSEs. Its construction protocol is based on “self-assembled matrix secretion” or “endogenous matrix secretion”. First, fibroblasts were seeded in medium containing ascorbic acid, and the dermal layer structure was formed using the cells’ own secretory function. After the dermal matrix stabilized, keratinocytes were seeded and transferred to air–liquid interface culture to induce stratum corneum differentiation. The core advantage of this model is that it can be maintained in vitro for up to 3 months, supporting the observation of the long-term pathological process of psoriasis and making it suitable for mechanism research requiring long-term tracking. The second type is Collagen matrix HSEs. It adopts a simplified protocol of “pre-fabricated matrix”. First, a cell-free collagen layer was constructed as the dermal scaffold, followed by sequential seeding of fibroblasts and keratinocytes. Epidermal differentiation was completed through air–liquid interface culture. A prominent feature of this model is its short preparation time, enabling rapid acquisition of equivalents with basic skin structure, while its in vitro maintenance period is only about 2 weeks, making it more suitable for the detection of short-term pathological phenotypes. Notably, these models lack immune cells and depend on exogenous cytokines to sustain the pathological environment. To address this limitation, the team further developed immune-competent HSEs: TH1-polarized CD4+ T cells were integrated into the skin equivalent structure, forming a “skin cell-immune cell” co-culture system, and the model function was validated through multi-dimensional detection. It achieved functional integration of immune cells with skin structure for the first time, spontaneously exhibiting psoriasis-like phenotypes without exogenous cytokines, which is closer to the pathological mechanism of “immune cell–keratinocyte interaction.” However, there are phenotypic differences between the TH1 cells used and the TH17 cells, the main pathogenic cells in psoriasis, which reduces the accuracy of pathological simulation to a certain extent.

Morin et al. focused on the clinical characteristic of high disease heterogeneity in psoriasis, developing personalized human skin equivalents based on patient-derived primary cells and provide a new tool for individualized therapy research [53]. Initially, patient-derived fibroblasts were cultured to secrete and form the extracellular matrix (ECM) as the dermal layer. Subsequently, keratinocytes were seeded and transferred to air–liquid interface culture to induce epidermal differentiation, ultimately forming a psoriasis skin equivalent model with patient-specific characteristics. This psoriasis model not only preserved the genetic and epigenetic characteristics of patient-derived cells, making it suitable for investigating individualized treatments, but also allows adjustment of medium composition to accurately investigate the pathogenic mechanism of target molecules in psoriasis. However, the model has two limitations: one is the lack of immune cells, failing to simulate the core pathological link of immune cell–keratinocyte crosstalk in psoriasis; the other is the limited sample size—only cells from 3 psoriasis patients were used, making it difficult to cover the disease heterogeneity of different genetic backgrounds and clinical subtypes.

In the context of drug development, the targeted 3D skin model constructed by Huth et al. achieves the integration of psoriasis inflammatory mechanism analysis and drug effect verification [54]. The team integrated normal human epidermal keratinocytes, dermal fibroblasts, and γδ-T cells with IL-23A responsiveness and IL-17A-producing capacity. First, keratinocytes and fibroblasts were used to generate a basic skin equivalent; after complete epidermal differentiation, γδ-T cells were injected into the dermal layer. The inflammatory cascade in the model was activated by adding IL-36γ to simulate the in vivo inflammatory initiation process. A combination of multiple technologies was used to detect the model’s inflammatory response and drug effects. Results showed that IL-36γ significantly upregulated the expression of IL-23A and IL-17A, while confirming the blocking mechanism of risankizumab. The advantage of this model lies in its strong targeting—it can be used to test the efficacy of drugs targeting the IL-23/IL-17 pathway and analyze the regulatory effect of drugs on the inflammatory feedback loop. However, the model has limitations: it only focuses on the IL-36γ-IL-23A-IL-17A pathway and does not cover other key inflammatory pathways (such as the TNF-α and IL-6 pathways). Additionally, the type of immune cells is single, lacking other immune cells involved in psoriasis such as Treg cells and neutrophils. Future research needs to further expand the scope of cell types and pathway studies.

#### 3.3.2. Atopic Dermatitis, AD

In exploring the pathological mechanisms and drug development for atopic dermatitis (AD), 3D skin models serve as a critical tool bridging in vitro studies and clinical applications by simulating key pathological features of AD, such as barrier defects and inflammatory responses. One core pathological characteristic of AD is immune dysregulation dominated by Th2-type inflammatory responses [69,70,71,72,73,74,75]. To recapitulate the inflammatory microenvironment of AD, some research teams have incorporated Th2 cytokines or inflammatory stimuli into these models, thereby supporting mechanistic investigations and drug screening. For example, Flori et al. utilized immortalized human keratinocyte cell lines to construct 3D human epidermal equivalents (HEEs) through air–liquid interface culture [55]. During the cultivation process, the team added Th2 cytokines and successfully recapitulated the two core pathological features of AD: firstly, lipid metabolism abnormalities, with the model exhibiting AD-associated disruptions in epidermal lipid composition; and secondly, barrier function defects, where the epidermal integrity and permeability aligned with AD pathological manifestations. Further drug testing confirmed that this model can effectively validate the therapeutic potential of tofacitinib for AD, providing a reliable platform for studying inflammatory mechanisms. However, HEEs contain only the epidermal layer and lack key dermal components critical to AD pathology—such as dermal immune cells, stromal cells—and the skin microbiome. Consequently, they cannot simulate complex pathological processes like epidermal–dermal interactions or microbiome-immune crosstalk. Additionally, the AD-related lipid composition in the model differs from that of native human skin. This discrepancy may result in incomplete simulation of barrier function, potentially impacting the precise analysis of AD barrier defect mechanisms. Arroyo et al. chose human foreskin keratinocytes as seed cells. After serial culture, Th2 cytokines were added to construct an AD inflammatory model [56]. The core value of this model lies in drug evaluation; research confirmed that the model can effectively reflect the regulatory effects of drugs such as FK-866 and Olaparib on AD inflammation and cell proliferation, providing an experimental carrier for the preliminary screening of AD therapeutic drugs. However, the expression levels of NAMPT protein in the model differ from the clinical characteristics of AD patients, potentially affecting the simulation of AD-related metabolic mechanisms. Additionally, the treatment time window and dose gradient of Th2 cytokines have not been systematically optimized, which may lead to insufficient stability of pathological phenotypes. It is necessary to dynamically monitor and adjust parameters to enhance clinical relevance to the real AD pathogenic environment.

Kordulewska et al. further optimized the model structure to construct an AD model more closely resembling the complete skin architecture [57]. The team strictly controlled cell quality, using only passages 3–7 of normal human epidermal keratinocytes and dermal fibroblasts to ensure an undifferentiated state and viability. The construction process involved two steps: first, forming a dermal scaffold by mixing dermal fibroblasts with collagen; second, seeding epidermal keratinocytes and completing epidermal differentiation through air–liquid interface cultivation. Lipopolysaccharide (LPS) or histamine was finally added to activate AD-related inflammatory responses. The results showed that after stimulation, the model exhibited AD pathological features such as impaired epidermal barrier function, and significantly upregulated expression of inflammatory factors as well as genes related to the TLR2 signaling pathway. This model provides a reliable mechanism verification platform for the development of TLR pathway antagonists/inhibitors in the treatment of AD, while it still has limitations. Firstly, it lacks key immune cells involved in AD pathology, making it unable to recapitulate cell–cell interaction mechanisms. Secondly, it relies solely on two stimulants—lipopolysaccharide and histamine—failing to cover the complex etiologies involving multiple pathogenic factors, and the comprehensiveness of pathological simulation needs to be improved.

Genetic factors are important risk factors for the development of AD, which can affect barrier function by interfering with epidermal lipid metabolism [76,77,78]. Targeting this mechanism, Blunder et al. constructed an AD model based on keratinocytes with different genetic backgrounds, providing a new perspective on the heterogeneous pathological mechanisms of AD [58]. The team obtained primary keratinocytes from three groups of individuals: healthy controls, AD patients without FLG mutations, and AD patients with FLG mutations. These cells were cultured to establish human epidermal equivalents (HEEs), which successfully recapitulated the epidermal pathological features of AD. However, the model exhibited results inconsistent with clinical observations in barrier function detection. When testing the permeability of HEEs to hydrophilic substances, no significant barrier dysfunction was observed, which is inconsistent with the prevalent barrier defects in clinical AD patients. Possible reasons for this discrepancy include three aspects: first, the model lacks a complete regulatory system for lipid metabolism, leading to insufficient simulation of the lipid barrier. Secondly, the detection only targeted hydrophilic substances without evaluating the permeability of lipophilic substances, which may miss some AD-related barrier function defects; finally, the small sample size may affect the reliability of statistical results, and the sample size needs to be expanded for further verification.

### 3.4. Aged Skin Model

Constructing aged skin models poses unique challenges, as aging is a process involving the cumulative effects of genetic, physiological, and environmental factors, accompanied by physiological alterations induced by multiple elements [79]. Traditional models struggle to fully recapitulate such long-term cumulative effects; however, 3D aged skin models can authentically reflect the cellular structural changes of aged skin. The newly developed 3D bioprinted aged skin models attempt to mimic age-related skin functional changes. These models need to specifically focus on characteristics such as alterations in extracellular matrix components, disorders of collagen structure, and degradation of skin barrier function [66,79]. Given that skin is composed of multiple cell types, rapidly replicating this complex phenomenon remains challenging.

**Figure 3:**
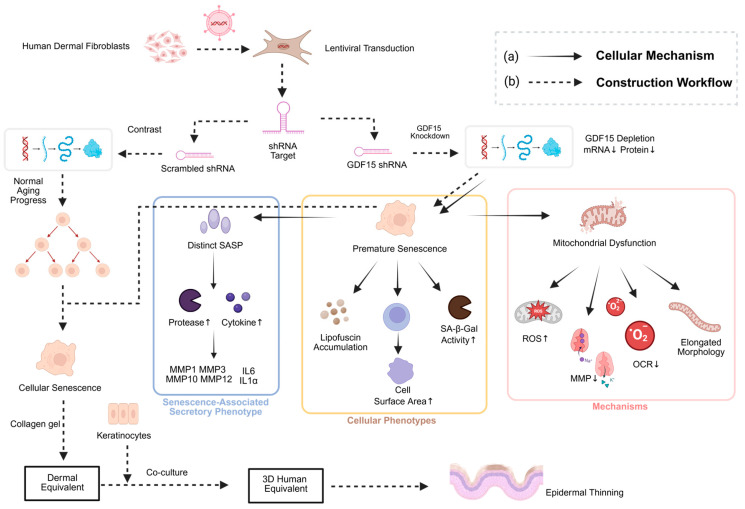
Mechanism and construction workflow of the GDF15-knockdown aged skin model. (a) Molecular mechanism according to Wedel et al. [59]: Lentiviral-mediated knockdown of GDF15 in human dermal fibroblasts triggers mitochondrial dysfunction, ROS accumulation, and the development of a Senescence-Associated Secretory Phenotype (SASP). (b) Tissue-level construction: Integration of these senescent fibroblasts into a 3D skin equivalent results in significant epidermal thinning, mimicking the characteristic atrophy observed in in vivo aged skin.

To investigate the role of growth differentiation factor 15 (GDF15) in maintaining mitochondrial function and delaying cellular senescence, Wedel et al. developed a 3D skin model to verify its association with aging, as illustrated in the mechanistic and construction workflow in Figure 3 [59]. The team used lentiviral transduction to introduce GDF15-targeting shRNA into dermal fibroblasts for GDF15 gene knockdown. This genetic modulation triggers mitochondrial dysfunction and ROS accumulation, leading to a distinct senescence-associated secretory phenotype (SASP) within the fibroblasts (Figure 3a). These fibroblasts were then mixed with collagen solution to induce polymerization, forming a gel-like 3D matrix as the dermal scaffold of the model. On the surface of the polymerized dermal layer, non-transduced normal epidermal keratinocytes were seeded and further cultured to form a complete skin equivalent structure (Figure 3b). The results showed that GDF15-knockdown fibroblasts led to a significant thinning of the model’s epidermis. This feature is highly consistent with the typical phenotype of epidermal atrophy during in vivo skin aging, confirming the key role of GDF15 in maintaining normal skin structure and delaying aging, and providing a functional verification platform for aging mechanism research.

Low et al. constructed a 3D aged skin model using human dermal fibroblasts and epidermal keratinocytes through multiple senescence induction methods, enhancing the model’s coverage of in vivo aging scenarios [60]. The team adopted three senescence induction strategies. First was replicative senescence, where cells enter a senescent state through continuous passaging. Second was drug/physical induction, namely the use of mitomycin C, doxorubicin, or ultraviolet B (UVB) to induce stress-induced senescence. Third was genetic induction, which directly triggers the cellular senescence program by overexpressing key senescence-associated genes such as p16INK4a or p14ARF. During preparation, pre-senescent-induced fibroblasts or keratinocytes were mixed with normal cells at a certain ratio to construct the model. First, fibroblasts were embedded in a collagen matrix to form the dermal layer, after which keratinocytes were seeded on the dermal layer for air–liquid interface culture to promote epidermal differentiation. Melanocytes or immune cells were added during the process to enhance model complexity. Model validation results showed that this 3D structure exhibited typical aging characteristics: reduced epidermal thickness, disrupted keratinocyte differentiation, damaged dermal structure, and significant impairment of skin barrier function, which is highly consistent with the pathological manifestations of aged skin.

3D aged skin models are effective tools for studying the relationship between cellular senescence and skin aging. They can partially simulate the structural and functional changes of aged skin and are used for drug screening. However, issues such as insufficient complexity, non-physiological induction methods, and limitations of biomarkers still require further optimization. In the future, more complex, multi-cell type, and dynamically regulatable model systems need to be developed to better simulate the in vivo aging process.

### 3.5. Skin Infection Model

In the field of skin infection mechanism research and anti-infection therapy development, recently developed 3D skin models have achieved a precise balance between the simplicity of construction, experimental reproducibility, and the complexity of wound pathological simulation, infection process reproduction, and therapeutic efficacy evaluation. They provide a more physiologically relevant in vitro platform for research in the skin infection field [61,80].

**Figure 4:**
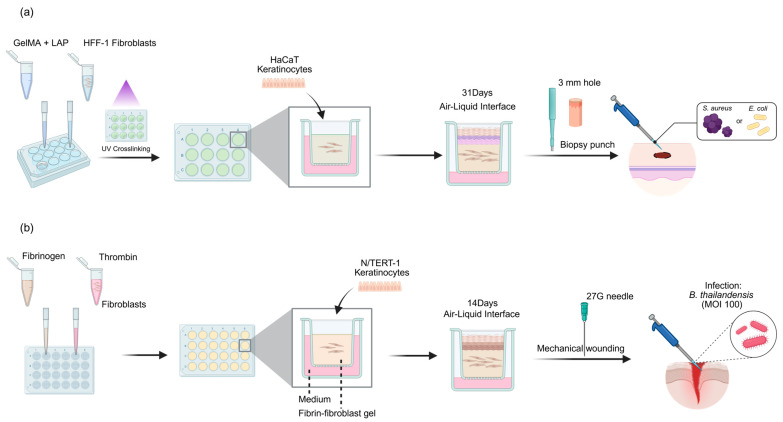
3D skin infection models for pathogen study and antibacterial screening. (**a**) GelMA-based biomanufacturing strategy (based on Villata et al. [61]): A UV-crosslinked hydrogel supports a complete skin structure for standardized infection with S. aureus or E. coli to evaluate the efficacy of antibacterial treatments. (**b**) Fibrin-based model construction (based on Ku et al. [62]): Human dermal fibroblasts and keratinocytes are cultured at the air–liquid interface (ALI), followed by mechanical wounding with a 27-gauge needle and inoculation with *Burkholderia thailandensis*.

Villata et al. developed a 3D in vitro skin model based on gelatin methacryloyl (GelMA) hydrogels, providing a standardized biomanufacturing strategy for antibacterial therapy screening (Figure 4a) [61]. The specific construction process involves preparing GelMA materials and seeding human dermal fibroblasts onto the hydrogel medium to form a 3D dermal layer. Subsequently, human keratinocytes are seeded on its surface for further culture to construct a complete epidermis–dermis composite structure. As shown in the biomanufacturing workflow (Figure 4a), the mature 3D skin model is then inoculated with Staphylococcus aureus (S. aureus) or Escherichia coli (E. coli) with an optical density (OD) value of 1.0. After 24 h of infection, a 5% penicillin–streptomycin (PS) mixed solution is used for antibacterial treatment to verify the effectiveness of the model in therapy evaluation. The 3D skin model constructed by this biomanufacturing strategy, with its excellent structural bionicity and experimental controllability, can be widely applied to the in vitro testing and efficacy verification of various anti-infection therapies.

In parallel, Ku et al. adopted a co-culture strategy involving a fibrin gel–fibroblast–keratinocyte matrix to construct a 3D skin equivalent model, as illustrated in the workflow in Figure 4b [62]. The core steps of its construction process are as follows: first, human dermal fibroblasts are embedded in the fibrin gel matrix to form a structure simulating the dermal matrix. Subsequently, human keratinocytes are seeded on the gel surface and continuously cultured for 14 days using the air–liquid interface (ALI) culture method to induce the differentiation and maturation of keratinocytes, forming a physiologically functional epidermal layer. After the model matures, a standardized wound is created in the epidermal layer using a 27-gauge needle, followed by the inoculation of target pathogens to establish a wound infection model (Figure 4b). With the help of this model, the study achieved an important discovery—it first revealed the host’s defense mechanism mediated by inflammasome activation to induce cell extrusion during Burkholderia infection. However, it also pointed out its limitations in model representativeness, quantification methods, and immune environment simulation.

## 4. Challenges in Clinical Translation

### 4.1. Lack of Model Standardization and Validation Systems

Currently, the primary challenge for 3D skin models in clinical translation is the absence of a unified standardization system and validation methods. Studies have shown that existing 3D culture systems face challenges in implementing standardized monitoring of culture conditions, which severely hinders the reproducibility of research results and their clinical translation [81]. During model construction, significant differences exist in cell sources, culture conditions, and evaluation indicators adopted by different laboratories, leading to a lack of comparability between models [82]. Especially in application scenarios such as skin cancer detection, existing datasets generally have issues of selection bias and insufficient standardization, limiting the clinical applicability of algorithm development [83]. In addition, inconsistencies in feature extraction and analysis methods across different software platforms have also delayed the clinical deployment of related technologies [33].

### 4.2. Technical Bottlenecks in Simulating Pathological Complexity

The complex structure of human skin poses significant technical challenges for the construction of disease models, and existing models still struggle to fully replicate the skin’s 3D structure and heterogeneous characteristics. Structurally, many models fail to fully recapitulate the skin’s full thickness—especially the integration of the subcutaneous layer, which is often overlooked—thereby limiting their ability to reproduce the features of skin diseases [4,84]. In addition, the skin’s 3D structure exhibits complex heterogeneity, including cell–cell signaling networks and microenvironmental differences. Existing methods cannot accurately reproduce these aspects, thus failing to fully recapitulate the pathological characteristics of skin diseases [2,4,18].

Integrating a functional vascular system is the core bottleneck of 3D vascularized skin models. Currently, it is not possible to effectively integrate vascular networks into the model to support cell survival and physiological functions. The difficulty lies in establishing perfusable vascular structures that simulate the physiological demands for nutrient and oxygen distribution. Meanwhile, the formation of vascular systems during bioprinting relies on complex bioink combinations (such as nanocomposite hydrogels and sacrificial materials). However, these methods tend to fail when constructing long-term sustainable vascular networks, leading to defects in model cell survival and metabolic activities that prevent the simulation of the nutrient delivery mechanism of real skin [47,85,86,87].

### 4.3. Establishment of Ethical Norms and Regulatory Frameworks

The clinical translation of 3D skin models also faces ethical and regulatory challenges, which are key factors driving model optimization. Although these models can reduce reliance on animal experiments, their clinical application still lacks sound ethical evaluation frameworks [12].

Ethical concerns are intertwined with technical limitations, jointly hindering the standardization and international application of the models. The core ethical issues involved include ethical matters related to tissue collection, ethical motivations for replacing animal models, and new ethical challenges brought about by stem cell technology. Currently, unified international standards for these issues have not been established. This is mainly reflected in ongoing discussions on ethical concerns such as donor rights and interests as well as source sustainability. Additionally, practical obstacles such as insufficient reproducibility and lack of standard protocols in the model development process lead to inconsistencies in global applications [88,89,90]. Furthermore, ethical norms need to be established to address data privacy and algorithm transparency issues arising from the application of AI-assisted diagnostic systems in dermatology [83].

### 4.4. The Balance Between Medical Costs and Benefits

The clinical application of 3D skin model technology still faces the challenge of balancing cost and benefit. While such models are expected to improve the accuracy of drug testing and personalized treatment, their high R&D and production costs may limit clinical promotion [12]. Studies have shown that ensuring the production of skin models with physiological relevance requires substantial resource investment in process optimization and quality control [38]. In environments with limited medical resources, balancing model complexity and accessibility has become a key issue—particularly the need to strike a balance between model simplicity and the reproduction of complex pathological characteristics such as wounds and infections [61]. Additionally, the lack of standardization in research methods when applying technologies like deep learning in dermatology has further increased the cost burden of clinical translation [24].

To optimize the cost–effectiveness ratio, a ‘fit-for-purpose’ stratification strategy is essential for clinical translation. Not all experimental questions require biologically exhaustive, full-thickness models. For instance, in high-throughput cytotoxicity screening or routine skin irritation testing, simplified epidermal equivalents or basic full-thickness models lacking complex appendages are often sufficient to generate regulatory-compliant data without the prohibitive costs of vascularization or neural integration [35,40]. Conversely, the inclusion of high-cost components—such as perfusable vasculature, autologous immune cells, or patient-specific tumor spheroids—should be reserved for specific contexts requiring systemic interaction analysis, such as drug bioavailability studies or complex inflammatory disease modeling [18,50]. By decoupling structural complexity from functional necessity, researchers can reduce manufacturing overheads for routine assays while concentrating resources on high-fidelity models for critical translational bottlenecks.

## 5. Conclusions

In recent years, 3D human skin model technology has achieved significant progress, with a series of breakthroughs ranging from basic construction technology to disease-specific modeling. Innovations in bioprinting technology have enabled models to better recapitulate the 3D structure and barrier function of skin, while the optimization of multicellular co-culture systems has significantly improved the models’ ability to replicate the skin microenvironment [91,92,93]. These technological advances have collectively driven the transformation of skin models from simple structural simulation to functional simulation—particularly notable breakthroughs in modeling fields such as wound repair, skin tumors, and inflammatory diseases [94,95]. Specifically, regarding current capabilities, 3D skin models have evolved from experimental prototypes into robust platforms for biomedical application. It is now possible to routinely fabricate standardized full-thickness skin equivalents that replicate the barrier function and mechanical properties of native tissue, serving as reliable alternatives to animal models for toxicity and irritation testing. Furthermore, by incorporating patient-derived cells and functional vascular networks, researchers can currently generate personalized disease models to predict individual drug responses and recapitulate complex pathological microenvironments—such as tumor invasion and immune-mediated inflammation—with a fidelity that traditional preclinical tools cannot achieve. It is worth noting that these technological breakthroughs are not isolated events but demonstrate a distinct cumulative effect: early basic construction technologies laid the foundation for subsequent pathological simulation, while the demand for disease-specific modeling has in turn promoted the refined development of construction technologies [4,66]. This virtuous cycle of technological iteration is accelerating the process of translating 3D skin models from laboratory research to clinical applications.

Meanwhile, the development of 3D skin models has underscored the importance of interdisciplinary collaboration—a trend that will become even more prominent in the future. The construction of skin models requires the integration of professional knowledge from multiple fields, including tissue engineering, materials science, cell biology, and microfluidics technology [35,96]. Their clinical application, in turn, involves disciplines such as clinical medicine, pharmacy, data science, and bioinformatics. Particularly in the context of precision medicine, the integrated analysis of multi-omics data has endowed skin models with new value [96,97,98,99]. This interdisciplinary collaboration is not only reflected at the technical level but also requires the participation of social science fields such as law and ethics in the Establishment of ethical norms and regulatory frameworks [66]. In the future, establishing institutionalized interdisciplinary cooperation platforms will become a key pathway to advancing the development of 3D skin models [100].

Modern medicine is moving toward precision medicine, and multi-omics data plays a key role in deciphering biological phenomena and disease mechanisms [101]. Future strategies should integrate multi-omics data—including genomics, transcriptomics, and proteomics—and combine them with artificial intelligence algorithms to develop more accurate predictive models for skin diseases. In particular, the advancement of single-cell multi-omics and spatial multi-omics technologies has provided new dimensions for skin disease research [91,102]. The emergence of data integration platforms such as MANAclust has enabled the fusion of clinical parameters with multi-omics data, offering technical support for precision medicine [88]. Such a multi-omics integration approach has already demonstrated application potential in diseases like colorectal cancer, where machine learning technologies analyze massive volumes of omics data to support precise medical decision-making [103].

In addition, closed-loop solutions for precision medicine require establishing a complete chain from disease modeling to treatment feedback. Multi-omics data integration provides a platform for understanding disease heterogeneity, molecular mechanisms, and developing personalized treatment strategies. Organoid models combined with multi-omics technologies can delve into the microenvironment and predict responses to targeted therapy [104,105]. Artificial intelligence-driven multi-omics analysis methods have achieved significant progress in early screening, diagnosis, and prognosis prediction [91]. The integration of epigenetic therapy with multi-omics technologies indicates a new direction for treatment, holding promise for more effective personalized treatment strategies [106]. However, realizing true closed-loop solutions requires addressing the technical challenges of data integration and analysis, as well as establishing corresponding ethical frameworks [94,107,108]. Ultimately, the convergence of these technologies heralds a new era of ‘digital–physical’ duality in dermatology. We anticipate that the next paradigm shift will emerge from the integration of AI-driven ‘digital twins’ with next-generation 4D bioprinting. This synergy will not only allow for virtual clinical trials that precede physical testing but also pave the way for smart, vascularized skin grafts capable of in situ dynamic adaptation. Such breakthroughs promise to bridge the final gap between benchside models and bedside applications, transforming 3D skin technology from a preclinical screening tool into a direct therapeutic modality for personalized regenerative medicine.

## Figures and Tables

**Table 1:** Comparison of Traditional Research Models vs. Advanced 3D Skin Models.

Model Type	Key Characteristics	Efficacy & Strengths	Limitations & Challenges
Traditional 2D Cell Culture	Monolayer of cells grown on flat plastic surfaces.	1. High Throughput: Simple, cost-effective, and easy to scale for drug screening. 2. Reproducibility: Well-established protocols with low variability.	1. Lack of Architecture: Fails to replicate the layered structure and barrier properties of native skin. 2. Poor Physiological Relevance: Cannot simulate complex cell–matrix interactions or 3D microenvironments
Animal Models	Use of laboratory animals (e.g., mice, pigs) to simulate human skin conditions.	1. Systemic Complexity: Includes intact immune, vascular, and nervous systems. 2. In Vivo Context: Captures whole-organism responses to drugs or injuries.	1. Interspecies Disparities: Significant differences in skin anatomy and healing mechanisms limit translation to humans. 2. Ethical & Cost Issues: High maintenance costs and increasing ethical controversies.
3D Skin Models	Bioengineered 3D constructs mimicking human skin architecture and function.	1. High Fidelity: Replicates the stratified epidermis–dermis structure and barrier functions of human skin. 2. Controlled Microenvironment: Enables precise manipulation of specific pathological factors.	1. Incomplete Systemic Integration: Generally lacks fully functional vascular networks and neural innervation. 2. Technical Complexity: High fabrication costs, lack of standardized validation protocols, and batch-to-batch variability.

**Table 2:** Summary of Strengths and Weaknesses of Skin Organoid Technology.

Aspect	Advantages	Challenges
Structural Complexity	1. High Architectural Fidelity 2. Appendage Formation 3. Barrier Formation	1. Incomplete Physiology 2. Stochastic Variability
Functional & Systemic Mimicry	1. Regeneration Potential 2. Immune Integration	1. Lack of Systemic Interaction 2. Maturation Issues
Disease Modeling & Applications	1. Personalized Medicine 2. Disease Dynamics 3. Ethical Superiority	1. Heterogeneity 2. Control Difficulty

**Table 3:** Summary of 3D skin models’ construction strategies, and outcomes.

Model Category	Reference	Construction Strategy/Methodology	Key Findings/Biological Outcomes
**Wound Healing**	**Zhang et al. [25]**	**Organoid + 3D Printing:** Combined skin organoids with extrusion bioprinting using dual photo-crosslinking technology.	**Accelerated Healing:** Significantly accelerated the closure of extensive wounds, achieving in situ regeneration, spontaneous microvascular network formation, and suppression of excessive inflammation.
	**Maggiotto et al. [43]**	**Vascularized Model:** Used GelMA (matrix) and Pluronic F127 (sacrificial ink) for multi-material bioprinting; incorporated HUVECs to build vascular channels.	**Functional Perfusion:** Endothelial cells successfully formed barrier-functional vascular structures; the model achieved spontaneous wound closure within 14 days, confirming regenerative potential under perfusion.
	**Chen et al. [44]**	**Spatiotemporal Biomimetic Model:** Three-layer structure (surface frame, middle hydrogel, bottom factor-releasing gel). Surface provides mechanical pre-tension; bottom releases factors.	**Dynamic Repair:** The frame-assisted contraction significantly shortened wound closure time, while controlled cytokine release promoted granulation tissue formation and re-epithelialization
	**Choi et al. [45]**	**DLP 3D Printing:** Used Silk-GMA and Gel-GMA hybrid ink supplemented with EGF (Epidermal Growth Factor).	**Long-term Stability:** Maintained structural integrity for over 4 weeks without degradation; EGF incorporation significantly enhanced epidermal regeneration rates and dermal repair quality.
	**Xu et al. [46]**	**Material Modification:** Introduced Halloysite Nanotubes (HNT) into Collagen–Alginate–Hyaluronic Acid hydrogel.	**Inflammation Modulation:** HNT reinforcement promoted keratinocyte differentiation and regulated inflammatory factor release, facilitating wound healing with reduced scar formation.
**Melanoma**	**Daugaard et al. [47]**	**Full-Thickness (FT) Co-culture:**	**Phenotypic Fidelity:** Melanoma cells retained invasive phenotypes and gene expression profiles; validated the efficacy of vemurafenib in reducing tumor spheroids within the 3D microenvironment.
		1. FT-Spheroids: Melanoma spheroids embedded in the dermis.	
		2. FT-EPI: Cells seeded directly on the dermal surface.	
	**Sandri et al. [48]**	**Drug Resistance Models:**	**Mechanism of Resistance:** Revealed that drug-resistant cells could breach the epidermal–dermal junction, driven by significantly upregulated MMP-2 expression compared to non-resistant cells
		1. Liquid Overlay: Spheroids in collagen matrix.	
		2. Air–Liquid Interface (ALI): Pigmented multi-layered tissue.	
	**Massaro et al. [49]**	**Invasion & Drug Response:**	**Anti-Invasive Screening:** Validated that 2-Methoxyestradiol (2-ME) significantly inhibited melanoma invasion; the model accurately simulated drug penetration profiles through skin tissue.
		1. Spheroid Implantation: For small molecule screening.	
		2. Full-Thickness Model: ALI culture mimicking in vivo state.	
**cSCC**	**Kurzyk et al. [50]**	**cSCC Heterogeneity Printing:** 3D bioprinting integrating HaCaT, adult fibroblasts, and A431 cancer cells.	**Therapeutic Validation:** Demonstrated complex cellular interactions superior to 2D/monoculture models; validated the efficacy of cetuximab monotherapy in a multicellular tumor microenvironment.
	**He et al. [51]**	**Full-Thickness Model (FTM):** Integrated Cancer-Associated Fibroblasts (CAFs).	**CAF-Mediated Efficacy:** Drug-loaded nanoparticles exhibited potent anti-tumor activity; revealed that modulating CAF function could further enhance the therapeutic efficacy of nanoparticle treatments.
**Psoriasis**	**Scheurer et al. [52]**	**Immune-Competent Model:**	**Spontaneous Pathology:** Achieved functional immune–skin integration, spontaneously exhibiting psoriasis-like phenotypes solely through immune cell–keratinocyte crosstalk without artificial cytokine stimulation.
		1. Fibroblast-derived matrix (maintained for 3 months).	
		2. Rapid collagen synthesis.	
		3. Integrated Th1-polarized CD4+ T cells.	
	**Morin et al. [53]**	**Personalized Model:** Used patient-derived primary cells (adult fibroblasts and keratinocytes).	**Patient Specificity:** Preserved donor-specific genetic and epigenetic characteristics, providing a precise platform for investigating individualized treatment responses.
	**Huth et al. [54]**	**Pathway-Targeted Model:** Integrated γδ-T cells; induced inflammation via IL-36γ.	**Pathway Blockade:** Confirmed that IL-36γ upregulation of IL-23A/IL-17A could be effectively blocked by risankizumab, validating the model for IL-23/IL-17 pathway-targeted drug testing.
**Atopic Dermatitis**	**Flori et al. [55]**	**Human Epidermal Equivalents (HEE):** Immortalized keratinocytes + Th2 cytokine stimulation.	**Lipid Abnormalities:** Reproduced AD-associated epidermal lipid composition defects and barrier dysfunction; validated the therapeutic efficacy of tofacitinib.
	**Arroyo et al. [56]**	**Inflammation & Drug Evaluation:** Primary cells + Th2 cytokines.	**Pharmacological Screening:** Successfully reflected the regulatory effects of drugs such as FK-866 and Olaparib on AD-related inflammation and cell proliferation.
	**Kordulewska et al. [57]**	**Full-Thickness Model:** Strictly controlled cell passage number + LPS/Histamine stimulation.	**TLR Activation:** Stimulation significantly upregulated inflammatory factors and TLR2 signaling pathway genes, validating the model for screening TLR antagonists
	**Blunder et al. [58]**	**Genetic Background Model:** Constructed HEE using cells from *FLG*-mutant patients.	**Genetic Phenotyping:** Recapitulated epidermal pathological features of FLG mutations; notably, hydrophilic permeability tests showed inconsistent barrier defects compared to clinical data, highlighting specific model limitations
**Aging Skin**	**Wedel et al. [59]**	**Gene Knockdown Model:** Silenced *GDF15* in fibroblasts using shRNA.	**Mechanism of Atrophy:** Confirmed that GDF15 depletion triggers mitochondrial dysfunction and ROS accumulation, leading to significant epidermal thinning characteristic of aged skin.
	**Low et al. [60]**	**Multi-Induction Model:** Strategies include replicative senescence, UV/drug induction, and gene overexpression (*p16*/*p14*).	**Comprehensive Aging Signs:** Successfully exhibited typical aging features including reduced epidermal thickness, disordered differentiation, and significant barrier function impairment
**Skin Infection**	**Villata et al. [61]**	**GelMA Hydrogel Model:** Inoculated with *Staphylococcus aureus* or *Escherichia coli*.	**Antibacterial Testing:** Established a standardized platform with high structural biomimicry; validated the efficacy of penicillin–streptomycin treatment against bacterial infection
	**Ku et al. [62]**	**Fibrin Hydrogel Model:** Physical wounding + *Candida albicans* inoculation.	**Host Defense Discovery:** First report of a host defense mechanism mediated by inflammasome activation that induces cell extrusion to eliminate pathogens

Abbreviations: GelMA, gelatin methacryloyl; HUVEC, human umbilical vein endothelial cells; EGF, epidermal growth factor; HNT, halloysite nanotubes; ALI, air–liquid interface; MMP-2, matrix metalloproteinase-2; cSCC, cutaneous squamous cell carcinoma; CAF, cancer-associated fibroblasts; HEE, human epidermal equivalents; LPS, lipopolysaccharide; TLR, Toll-like receptor; GDF15, growth differentiation factor 15; FT, full-thickness.

## Data Availability

No new data were created or analyzed in this study.

## Abbreviations

2D	Two-dimensional
3D	Three-dimensional
AD	Atopic dermatitis
AI	Artificial intelligence
ALI	Air–liquid interface
CAF	Cancer-associated fibroblast
CRISPR/Cas9	Clustered regularly interspaced short palindromic repeats/CRISPR-associated protein 9
cSCC	Cutaneous squamous cell carcinoma
EGF	Epidermal growth factor
FLG	Filaggrin
FT	Full-thickness
GDF15	Growth differentiation factor 15
GelMA	Gelatin methacryloyl
HEE	Human epidermal equivalent
HNT	Halloysite nanotube
HUVEC	Human umbilical vein endothelial cell
LPS	Lipopolysaccharide
MMP-2	Matrix metalloproteinase-2
shRNA	Short hairpin RNA
TLR	Toll-like receptor
UV	Ultraviolet
